# Joint Active and Passive Beamforming in RIS-Assisted Secure ISAC Systems

**DOI:** 10.3390/s24010289

**Published:** 2024-01-03

**Authors:** Jinsong Chen, Kai Wu, Jinping Niu, Yanyan Li

**Affiliations:** 1 School of Information Science and Technology, Northwest University, Xi’an 710127, China; 202121822@stumail.nwu.edu.cn (J.C.); jinpingniu@nwu.edu.cn (J.N.); liyanyan@nwu.edu.cn (Y.L.); 2 Global Big Data Technologies Centre (GBDTC), University of Technology Sydney, Sydney, NSW 2122, Australia

**Keywords:** reconfigurable intelligent surfaces (RIS), integrated sensing and communications (ISAC), physical-layer secrecy

## Abstract

This paper investigates joint beamforming in a secure integrated sensing and communications (ISAC) system assisted by reconfigurable intelligent surfaces (RIS). The system communicates with legitimate downlink users, detecting a potential target, which is a potential eavesdropper attempting to intercept the downlink communication information from the base station (BS) to legitimate users. To enhance the physical-layer secrecy of the system, we design and introduce interference signals at the BS to disrupt eavesdroppers’ attempts to intercept legitimate communication information. The BS simultaneously transmits communication and interference signals, both utilized for communication and sensing to guarantee the sensing and communication quality. By jointly optimizing the BS active beamformer and the RIS passive beamforming matrix, we aim to maximize the achievable secrecy rate and radiation power of the system. We develop an effective scheme to find the active beamforming matrix through fractional programming (FP) and semi-definite programming (SDP) techniques and obtain the RIS phase shift matrix via a local search technique. Simulation results validate the effectiveness of the proposed methods in enhancing communication and sensing performance. Additionally, the results demonstrate the effectiveness of introducing the interference signals and RIS in enhancing the physical-layer secrecy of the ISAC system.

## 1. Introduction

With the explosive growth of wireless devices, spectrum and energy resources have become a valuable asset. The scarcity of these resources has promoted the development of more efficient technologies. Integrated sensing and communications (ISAC) is a promising technology to substantially enhance the spectral and energy efficiency of numerous next-generation wireless systems [1,2]. Due to their unique integration and coordination advantages, ISAC systems have attracted widespread interest in various fields [3], such as vehicle networks, unmanned aerial vehicle sensing, and communication and localization sensing, etc. In ISAC systems, many efforts have been devoted to designing ISAC waveforms to achieve the simultaneous support of target detection and communications through simultaneous sensing and communications [4,5,6,7]. A main focus has been on transmitting designs for enhancing the communication performances of legitimate users, with limited attention to the presence of potential eavesdroppers in the environment.

From the perspective of typical radar systems, the power of the sensing signal can be concentrated in the direction of targets of interest to ensure high detection performance. For the ISAC system, the transmitting signal encompasses not only sensing information but also communication information. Therefore, the communication information is susceptible to being intercepted by potential sensing targets, and these sensing targets are likely to be potential eavesdroppers, introducing potential security risks into the ISAC system. To tackle the security issues in the ISAC system, potential eavesdroppers can be regarded as radar targets [8]. While preventing eavesdropping on legitimate information, it is also essential to ensure the system maintains optimal sensing performance. However, the challenge lies in achieving a balance between suppressing eavesdropping and ensuring satisfactory sensing performance. The pursuit of robust sensing performance may inadvertently lead to sub-optimal communication performance for legitimate users in the ISAC system.

Various schemes have been proposed to address the security issue and maximize the communication security rate, such as artificial noise interference and multi-antenna beamforming [9,10,11,12,13,14,15], etc. In [12], the security of ISAC was investigated, aiming to maximize the signal-to-interference-plus-noise ratio (SINR) of the radar while ensuring the achievement of secure rates for legitimate users. The authors in [13] proposed an auxiliary method for Artificial Noise (AN) deployment in an ISAC system. This approach involves the base station (BS) providing communication services to legitimate users while concurrently detecting radar targets. In [14], pseudo-random interference signals were introduced during the transmission of communication signals. These signals are designed to disrupt eavesdroppers’ attempts to intercept useful signals and simultaneously act as signals for detecting targets. In [15], a multiple-user interference was leveraged to address security issues in the dual-functional radar and communication (DFRC) system. Constructive interference is employed to enhance the received signal at communication users, while destructive interference is utilized to degrade eavesdropping signals at radar targets. In the context of the ISAC system, it is crucial to enhance the communication secrecy rate for legitimate users while maintaining sensing performance. But, for the secure communications in the ISAC system, the performance is heavily constrained by the wireless propagation environment.

Recently, due to the introduction of RIS to beyond 5G communications, RIS-based ISAC has attracted extension attention. For instance, in [16], RIS is employed in an ISAC system to enhance downlink communications. This is achieved by maximizing SINR for radar applications and minimizing the multi-user interference (MUI) for communication purposes. In [17], RIS is employed to mitigate MUI under the Cramer–Rao bound (CRB) constraint for direction of arrival (DOA) estimation. In [18], a study on fair sensing–communication waveform design with RIS is conducted. In this study, the joint optimization of beamforming at the BS and RIS is performed. The optimization aims to maximize the sensing SINR and minimize the MUI for communication.

Given the potential of RIS in improving ISAC performances, RIS has also become popular in secure ISAC designs. In [19], the secrecy rate of legitimate users was enhanced through the introduction of RIS. In [20], RIS was used to sense and locate targets in wireless networks, where a special sensor is installed near the RIS to sense the direction of nearby targets through the RIS. In [21], RIS was used to assist the wireless communication system of a security classification to ensure the quality of service of ordinary users and the safe rate of confidential users while reducing the transmission power of the BS. In [22], RIS was employed to assist the ISAC system. This involves maximizing the output SINR of the radar while ensuring the quality of service (QoS) of communication. In [23], by jointly designing the radar’s received beamformer, active RIS reflection coefficient matrix, and transmit beamforming matrix, the maximum secrecy of the system was achieved under the conditions of the total power budget and the minimum signal-to-noise-ratio (SNR) of the radar. In [24], RIS was employed to assist the communication link between the BS and legitimate users while simultaneously aiding in the detection of obstructed sensing targets. The objective is to maximize the secrecy rate for legitimate users while ensuring a specified SINR for sensing. From these works, we see that RIS can provide additional communication links to improve the performance of communication networks, while increasing the SINR of legitimate users while suppressing the SINR of eavesdroppers. At the same time, RIS has expanded the ISAC system coverage range, not only ensuring communication performance, but also improving sensing performance. Inspired by the aforementioned efforts, we intend to leverage RIS to maximize both communication secrecy rate and radar radiation power towards a target.

In this paper, we leverage the potential of RIS to modify the wireless environment and design an ISAC transmitting waveform to improve the performance of the RIS-assisted secure ISAC system. We formulate an optimization problem to maximize the secrecy rate for legitimate users and the radar radiation power towards potential eavesdroppers. The main contributions are summarized as follows:

- In order to suppress the eavesdropper from intercepting legitimate users’ information, the BS is designed to simultaneously transmit communication signals and interference signals, which is to achieve both sensing and communication by introducing the designed interference signals into the system. To maximize the communication secrecy rate and radar radiation power, we jointly optimize the communication beamformer, interference signal beamformer, and the reflection coefficient matrix of the RIS. Specifically, under power and phase constraints, we maximize the secrecy rate in logarithmic form and maximize the detection power in quadratic form, rendering a highly non-convex problem that is challenging to solve.
- We reformulate the secrecy rate problem as a fractional programming (FP) problem. Together with maximizing the radiation power, we then cast the problem into a semi-definite programming (SDP) formulation. The combined use of FP and SDP addresses the challenges posed by the multi-ratio fractional and non-convex optimization aspects of the problem. This enables us to further apply the semi-definite relaxation (SDR) for solving the reformulated optimization problem. Subsequently, we employ an alternating optimization framework to optimize the active beamforming matrix and the reflection coefficient matrix to achieve the final solution.

The remainder of this paper is organized as follows. Section 2 introduces the system model of the considered RIS-assisted secure ISAC system. Section 3 introduces the problem formulation involved in the system, as well as develops the joint beamforming scheme. Section 4 presents and discusses the numerical and simulation results, and Section 5 concludes the paper.

*Notations:* Bold lowercase letters and bold uppercase letters denote column vectors and matrices, respectively. C represents the set of complex numbers. ${∥\cdot ∥}^{2}$ denotes the Euclidean norm, and ${∥\cdot ∥}_{F}$ denotes the Frobenius-norm of its argument. $\mathrm{diag}(a_{1},a_{2},\ldots ,a_{N})$ denotes an *N*-dimensional diagonal matrix whose diagonal elements are $a_{1},a_{2},\ldots ,a_{n}$. ${\left\{\cdot \right\}}^{T}$ and ${\left\{\cdot \right\}}^{H}$ and denote the transpose and conjugate transpose operation, respectively. $I_{M}$ represents the M×M identity matrix. ${\left\{\cdot \right\}}^{*}$ is the conjugate operation. $E\left\{\cdot \right\}$ is the expectation operation. $ℜ\left\{\cdot \right\}$ is the real part of a complex number.

## 2. System Model

**Figure 1:**
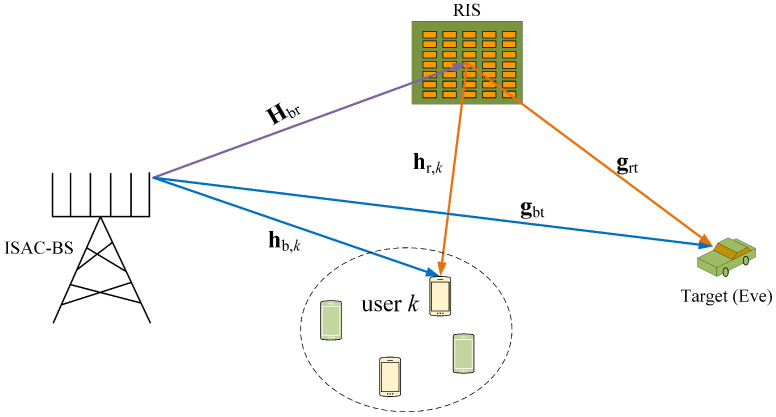
A RIS-aided secure ISAC system.

We consider a secure RIS-aided ISAC system that includes a dual-functional BS, a RIS, an eavesdropper that can be treated as a sensing target, and *K* single antenna users, as illustrated in Figure 1. The BS is equipped with a uniform linear array of *M* transmitting antennas, serving *K* users (M≥K) while detecting the target. The RIS is with *N* reflecting elements, each of which has a discrete adjustable phase. Let Θ be the diagonal reflecting phase matrix of the RIS, $\Theta =\mathrm{diag}\left\{\phi_{1},\cdots ,\phi_{n},\cdots ,\phi_{N}\right\}$, and $\phi_{n}=e^{j\vartheta_{n}}$, where $\vartheta_{n}=\frac{2\pi i}{2^{b}},i=\left\{0,1,\ldots ,2^{b}-1\right\}$ is the set of possible phase values for the *n*-th RIS element and *b* is the number of quantization bits. The users are uniformly distributed within a confined area, while the potential target is located in a different area.

This work focuses on designing the ISAC waveform for different stages of the physical-layer secrecy enhancement. First, we consider refining the target detection based on prior knowledge of the coarse direction of the target, ensuring communication quality of service for legitimate users in the meantime. In the second stage, we design ISAC waveforms to actively transmit interference signals towards eavesdroppers, enhancing the secrecy rate and further improving target detection. To achieve this goal, the BS is designed to simultaneously transmit two different signals, the communication signal $s_{c}$ and interference signal $s_{r}$, and both signals can be used to detect the potential target. Assume that both $s_{c}$ and $s_{r}$ are simultaneously transmitted for communications and sensing by the shared antennas, where $s_{c}$ contains information required by legitimate users, and $s_{r}$ is used to interfere with the eavesdropper. We assume that the prior location information of the eavesdropper has been obtained. In the ISAC system, such information can be achieved by the sensing function and treating the eavesdropper as a sensing target [15,24]. Let $W_{c}\in C^{M\times K}$ represent the related communication beamformer and $W_{r}\in C^{M\times M}$ denote the corresponding interference beamformer. Let x be the dual-function signal from the BS, and x can be expressed as

(1) $$\begin{array}{c} x=W_{c}s_{c}+W_{r}s_{r}, \end{array}$$

where $s_{c}\in C^{K\times 1}$ and $s_{r}\in C^{M\times 1}$. To avoid mutual interference between communication and interference signals, we assume that the communication and interference signals are statistically independent and uncorrelated [23], i.e., $E\left\{s_{c}s_{r}^{H}\right\}=0$. This is a typical and legitimate assumption in the sense that both signals are noise-like in the time domain and generated independently. And the communication and interference signals satisfy $E\left\{s_{c}s_{c}^{H}\right\}=I_{K}$ and $E\left\{s_{r}s_{r}^{H}\right\}=I_{M}$, where $I_{X}$ represents an *X*-order identity matrix. For convenience, we introduce $W=\left[W_{c},W_{r}\right]\in C^{M\times (K+M)}$ and $s={\left[s_{c}^{T},s_{r}^{T}\right]}^{T}\in C^{1\times (K+M)}$.

### 2.1. Communication Model

Let $H_{\mathrm{br}}\in C^{N\times M}$ denote the channel information matrix from the BS to the RIS, $h_{b,k}^{H}\in C^{1\times M}$ denote the channel vector from the BS to user *k*, and $h_{r,k}^{H}\in C^{1\times N}$ denote the channel vector from the RIS to user *k*. Based on the symbols and channels modeled above, the signal received at user *k* can be written as

(2) $$\begin{array}{c} y_{k}=\left(h_{b,k}^{H}+h_{r,k}^{H}\Theta H_{\mathrm{br}}\right)x+n_{k}, \end{array}$$

where $n_{k}$ is the additive white Gaussian noise (AWGN) with $n_{k}∼\mathrm{CN}\left(0,\sigma_{k}^{2}\right)$.

Denote $h_{\mathrm{br},k}=h_{b,k}^{H}+h_{r,k}^{H}\Theta H_{\mathrm{br}}$ as the composite communication channel from the BS to user *k*. Then, the received SINR at the *k*-th user can be written as

(3) $$\begin{array}{cc} \gamma_{k}= & \frac{{∥\left(h_{b,k}^{H}+h_{r,k}^{H}\Theta H_{\mathrm{br}}\right)w_{k}∥}^{2}}{\sum_{j=1,j\neq k}^{K+M}{∥\left(h_{b,k}^{H}+h_{r,k}^{H}\Theta H_{\mathrm{br}}\right)w_{j}∥}^{2}+\sigma_{k}^{2}}\\ = & \frac{{∥h_{\mathrm{br},k}w_{k}∥}^{2}}{\sum_{j=1,j\neq k}^{K+M}{∥h_{\mathrm{br},k}w_{j}∥}^{2}+\sigma_{k}^{2}}, \end{array}$$

where $w_{j}\in C^{M\times 1}$ represents the *j*-th column of W, and $\sigma_{k}^{2}$ denotes the noise power at the *k*-th user. According to (3), the achievable sum data rate of the legitimate users can be given by

(4) $$\begin{array}{c} R_{c}=\sum_{k=1}^{K}\log_{2}\left(1+\gamma_{k}\right). \end{array}$$

### 2.2. Sensing Model

Let $g_{\mathrm{bt}}=\mu_{\mathrm{bt}}a\left(\theta_{\mathrm{bt}}\right)\in C^{M\times 1}$ denote the direct channel from the BS to the target and $g_{\mathrm{rt}}=\mu_{\mathrm{rt}}a\left(\theta_{\mathrm{rt}}\right)\in C^{N\times 1}$ denote the channel from the RIS to the target. $\mu_{\mathrm{bt}}$ and $\mu_{\mathrm{rt}}$ denote the path loss from the BS to the target and from the RIS to the target, respectively. $\theta_{\mathrm{bt}}$ and $\theta_{\mathrm{rt}}$ denote the target direction from BS and RIS, respectively; let $a\left(\theta_{\mathrm{bt}}\right)={\left[1,e^{j2\pi \Delta \sin(\theta_{\mathrm{bt}})},\ldots ,e^{j2\pi \left(M-1\right)\Delta \sin\left(\theta_{\mathrm{bt}}\right)}\right]}^{T}\in C^{M\times 1}$ and $a\left(\theta_{\mathrm{rt}}\right)={\left[1,e^{j2\pi \Delta \sin(\theta_{\mathrm{rt}})},\ldots ,e^{j2\pi \left(N-1\right)\Delta \sin\left(\theta_{\mathrm{rt}}\right)}\right]}^{T}\in C^{N\times 1}$ be the steering vectors from the BS to the target and from the RIS to the target, respectively. Then, the radiation power in the direction of the target can be given by

(5) $$\begin{array}{cc} P_{b}= & {\left(g_{\mathrm{bt}}^{H}+g_{\mathrm{rt}}^{H}\Theta H_{\mathrm{br}}\right)}^{H}WW^{H}\left(g_{\mathrm{bt}}^{H}+g_{\mathrm{rt}}^{H}\Theta H_{\mathrm{br}}\right)\\ = & h_{\mathrm{bt}}^{H}WW^{H}h_{\mathrm{bt}}, \end{array}$$

where $h_{\mathrm{bt}}=g_{\mathrm{bt}}^{H}+g_{\mathrm{rt}}^{H}\Theta H_{\mathrm{br}}$ in (5).

In the considered ISAC system, the target is a potential eavesdropper, who attempts to decode the information sent to the legitimate users. The received SINR at the target in terms of the *k*-th user can be given by

(6) $$\begin{array}{cc} \gamma_{t,k}= & \frac{{∥h_{\mathrm{bt}}w_{k}∥}^{2}}{\sum_{j=1,j\neq k}^{K+M}{∥h_{\mathrm{bt}}w_{j}∥}^{2}+\sigma_{t}^{2}}, \end{array}$$

where $\sigma_{t}^{2}$ denotes the noise power at the target.

Based on (6), the achievable rate of the communication information eavesdropped by the target can be given by

(7) $$\begin{array}{c} R_{t}=\sum_{k=1}^{K}\log_{2}\left(1+\gamma_{t,k}\right). \end{array}$$

Then, the secrecy rate of the considered system can be computed by

(8) $$\begin{array}{cc} R_{s}= & R_{c}-R_{t}\\ = & \sum_{k=1}^{K}\log_{2}\left(1+\gamma_{k}\right)-\sum_{k=1}^{K}\log_{2}\left(1+\gamma_{t,k}\right). \end{array}$$

## 3. Problem Formulation and Algorithm Design

In this paper, we jointly design the BS transmit beamformer W and RIS phase shift matrix Θ to maximize the secrecy rate of the system and the radiation power of the target.

### 3.1. Problem Formulation

The problem is formulated as

(9) $$\begin{array}{ll} \max_{W,\Theta } & \rho R_{s}+P_{b}\\ s.t. & C1:{∥W∥}_{F}^{2}\leq P,\\ & C2:\vartheta_{n}=\frac{2\pi i}{2^{b}},i=\left\{0,1,\ldots ,2^{b}-1\right\}, \end{array}$$

where ρ is the regularization parameter, $P_{b}$ is the radiation power in the direction of the target (5), and *P* is the maximum transmission power of the BS. According to (3)–(6), the impact of W and Θ on the secrecy rate and radiation power optimization mainly depends on $\gamma_{k}$, $\gamma_{t,k}$, and $P_{b}$.

Problem (9) is a non-convex optimization problem and is difficult to solve directly. Next, we will first transform the problem into two optimization problems with respect to W and Θ, respectively, and then employ FP [25] and SDP to develop a low complexity solution to the original problem.

### 3.2. Proposed Scheme

In this section, we solve Problem (9) in two steps: optimizing W with a given RIS phase matrix and optimizing Θ with a fixed BS beamforming matrix. The two steps alternate and iterate until convergence.

#### 3.2.1. Step 1: Optimizing Active Beamforming Matrix W at the BS

We solve the BS beamforming matrix W by fixing the reflection coefficients matrix of the RIS, Θ. Then, Problem (9) can be simplified as

(10) $$\begin{array}{cc} \max_{W} & \rho \left(R_{c}-R_{t}\right)+P_{b}\\ s.t. & C1. \end{array}$$

Problem (10) is still non-convex, and we will transform it into a convex problem to find its solution. For the non-convex term involving the difference between two logarithmic functions in the first term, i.e., $\rho R_{s}=\rho \left(R_{c}-R_{t}\right)$, we apply the FP method [25] to recast it to a convex problem. For the second term $P_{b}$, we will reshape it to be convex, adopting a similar approach to that in [26]. By rephrasing the two terms, we rewrite the objective function with respect to the optimization variable W into a convex form.

We start with recasting $R_{c}$ in the objective function of (10) as a convex form regarding W. According to (4), $R_{c}$ in (8) can be expressed as

(11) $$\begin{array}{c} R_{c}=\sum_{k=1}^{K}\log_{2}\left(1+\frac{{∥h_{\mathrm{br},k}w_{k}∥}^{2}}{\sum_{j=1,j\neq k}^{K+M}{∥h_{\mathrm{br},k}w_{j}∥}^{2}+\sigma_{k}^{2}}\right). \end{array}$$

Applying the quadratic transformation [27], (11) can be expressed as a Lagrangian dual expression as

(12) $$\begin{array}{c} R_{c}=\sum_{k=1}^{K}\log_{2}\left(1+\beta_{k}\right)-\sum_{k=1}^{K}\beta_{k}+\sum_{k=1}^{K}\frac{\left(1+\beta_{k}\right){∥h_{\mathrm{br},k}w_{k}∥}^{2}}{\sum_{j=1,j\neq k}^{K+M}{∥h_{\mathrm{br},k}w_{j}∥}^{2}+\sigma_{k}^{2}}, \end{array}$$

where $\left[\beta_{k},\cdots ,\beta_{K}\right]$ is the auxiliary variable introduced. Note that there is still a fractional part on the right side of (12); hence, we apply the quadratic transformation again, leading to

(13) $$\begin{array}{cc} R_{c}= & \sum_{k=1}^{K}\log_{2}\left(1+\beta_{k}\right)-\sum_{k=1}^{K}\beta_{k}-\sum_{k=1}^{K}{\left|\varepsilon_{k}\right|}^{2}\sigma_{k}^{2}+2\sum_{k=1}^{K}\sqrt{1+\beta_{k}}ℜ\left\{\varepsilon_{k}^{*}h_{\mathrm{br},k}w_{k}\right\}\\ \; & -\sum_{k=1}^{K}{\left|\varepsilon_{k}\right|}^{2}\sum_{j=1,j\neq k}^{K+M}{\left|h_{\mathrm{br},k}w_{j}\right|}^{2}, \end{array}$$

where $\left[\varepsilon_{k},\cdots ,\varepsilon_{K}\right]$ is the auxiliary variable introduced.

Note that $\beta_{k}$ and $\varepsilon_{k}$ are auxiliary variables. With all other variables fixed, then $\beta_{k}$ and $\varepsilon_{k}$ can be obtained by solving the equations of $\frac{\partial R_{c}}{\partial \beta_{k}}$ and $\frac{\partial R_{c}}{\partial \varepsilon_{k}}$ equal to zero, respectively. With the details suppressed, $\beta_{k}$ and $\varepsilon_{k}$ are obtained as

(14) $$\begin{array}{c} \beta_{k}=\frac{{∥h_{\mathrm{br},k}w_{k}∥}^{2}}{\sum_{j=1,j\neq k}^{K+M}{∥h_{\mathrm{br},k}w_{j}∥}^{2}+\sigma_{k}^{2}}; \end{array}$$

(15) $$\begin{array}{c} \varepsilon_{k}=\frac{\sqrt{1+\beta_{k}}h_{\mathrm{br},k}w_{k}}{\sum_{j=1,j\neq k}^{K+M}{∥h_{\mathrm{br},k}w_{j}∥}^{2}+\sigma_{k}^{2}}. \end{array}$$

Define $\bar{w}=\mathrm{vec}\left\{W\right\}$ and $w_{j}=\Gamma_{j}\bar{w}$, where $\Gamma_{j}$ refers to a permutation matrix. Further define v and U as

(16) $$\begin{array}{c} v=\left[2\varepsilon_{k}^{*}\sqrt{1+\beta_{1}}h_{\mathrm{br},1},\cdots ,2\varepsilon_{k}^{*}\sqrt{1+\beta_{K}}h_{\mathrm{br},k},\cdots ,\right.{\left.0_{1\times M(K+M)-MK}\right]}^{H}\in C^{M(K+M)\times 1}, \end{array}$$

(17) $$\begin{array}{c} U={\left[u_{1,1},\cdots ,u_{K,K+M}\right]}^{T}\in C^{\left(K+M)\times M(K+M\right)}, \end{array}$$

where $u_{k,j}=\left|\varepsilon_{k}\right|\Gamma_{j}^{T}h_{\mathrm{br},k}^{H}$. Based on (13)–(17), $R_{c}$ can be further written into

(18) $$\begin{array}{c} R_{c}=f_{1}+ℜ\left\{v^{H}\bar{w}\right\}-{∥U\bar{w}∥}^{2}, \end{array}$$

where $f_{1}=\sum_{k=1}^{K}\log_{2}\left(1+\beta_{k}\right)-\sum_{k=1}^{K}\beta_{k}-\sum_{k=1}^{K}{\left|\varepsilon_{k}\right|}^{2}\sigma_{k}^{2}$.

Then, we recast $R_{t}$ in the objective function of (10) as a convex form regarding W. Similarly, $R_{t}$ in (8) can be expressed as

(19) $$\begin{array}{c} R_{t}=\sum_{k=1}^{K}\log_{2}\left(1+\frac{{∥h_{\mathrm{bt}}w_{k}∥}^{2}}{\sum_{j=1,j\neq k}^{K+M}{∥h_{\mathrm{bt}}w_{j}∥}^{2}+\sigma_{k}^{2}}\right). \end{array}$$

Its polynomial form can be given by

(20) $$\begin{array}{cc} R_{t}= & \sum_{k=1}^{K}\log_{2}\left(1+\beta_{r,k}\right)-\sum_{k=1}^{K}\beta_{r,k}-\sum_{k=1}^{K}{\left|\varepsilon_{r,k}\right|}^{2}\sigma_{t}^{2}+2\sum_{k=1}^{K}\sqrt{1+\beta_{r,k}}ℜ\left\{\varepsilon_{r,k}^{*}h_{\mathrm{br},k}w_{k}\right\}\\ \; & -\sum_{k=1}^{K}{\left|\varepsilon_{r,k}\right|}^{2}\sum_{j=1,j\neq k}^{K+M}{\left|h_{\mathrm{br},}\;kw_{j}\right|}^{2}, \end{array}$$

where $\beta_{r}$ and $\varepsilon_{r}$ are auxiliary variables and are given by

(21) $$\begin{array}{c} \beta_{r,k}=\frac{{∥h_{\mathrm{bt}}w_{k}∥}^{2}}{\sum_{j=1,j\neq k}^{K+M}{∥h_{\mathrm{bt}}w_{j}∥}^{2}+\sigma_{t}^{2}}; \end{array}$$

(22) $$\begin{array}{c} \varepsilon_{r,k}=\frac{\sqrt{1+\beta_{r,k}}h_{\mathrm{bt}}w_{k}}{\sum_{j=1,j\neq k}^{K+M}{∥h_{\mathrm{bt}}w_{j}∥}^{2}+\sigma_{t}^{2}}. \end{array}$$

Note that $\beta_{r,k}$ in (21) and $\varepsilon_{r,k}$ in (22) can again be obtained by solving the equations of $\frac{\partial R_{t}}{\partial \beta_{r,k}}$ and $\frac{\partial R_{t}}{\partial \varepsilon_{r,k}}$ equal to zero.

Define v1 and U1 as follows

(23) $$\begin{array}{cc} v1= & \left[2\varepsilon_{r,k}^{*}\sqrt{1+\beta_{r,1}}h_{\mathrm{bt}},\cdots ,2\varepsilon_{r,k}^{*}\sqrt{1+\beta_{r,K}}h_{\mathrm{bt}},\cdots ,\right.{\left.0_{1\times M(K+M)-MK}\right]}^{H}\\ & \in C^{M(K+M)\times 1}, \end{array}$$

(24) $$\begin{array}{c} U1={\left[u1_{1,1},\cdots ,u1_{K,K+M}\right]}^{T}\in C^{\left(K+M)\times M(K+M\right)}, \end{array}$$

where $u1_{k,j}=\left|\varepsilon_{r,k}\right|\Gamma_{j}^{T}h_{\mathrm{bt}}^{H}$. Based on (20)–(24), $R_{t}$ can be further written into

(25) $$\begin{array}{c} R_{t}=f_{2}+ℜ\left\{v1^{H}\bar{w}\right\}-{∥U1\bar{w}∥}^{2}, \end{array}$$

where $f_{2}=\sum_{k=1}^{K}\log_{2}\left(1+\beta_{r,k}\right)-\sum_{k=1}^{K}\beta_{r,k}-\sum_{k=1}^{K}{\left|\varepsilon_{r,k}\right|}^{2}\sigma_{t}^{2}$.

Substituting (18) and (25) into (8), $R_{s}$ becomes

(26) $$\begin{array}{cc} R_{s}= & f_{1}+ℜ\left\{v^{H}\bar{w}\right\}-{∥U\bar{w}∥}^{2}-f_{2}-ℜ\left\{v1^{H}\bar{w}\right\}+{∥U1\bar{w}∥}^{2}. \end{array}$$

Finally, we recast the last term $P_{b}$ in the objective function of (10) as a convex form regarding W. To make the last term convex, we first reformulate (5) as follows

(27) $$\begin{array}{cc} P_{b}= & h_{\mathrm{bt}}^{H}WW^{H}h_{\mathrm{bt}}\\ = & \sum_{k=1}^{K+M}w_{k}^{H}\left(MI_{M}-h_{\mathrm{bt}}h_{\mathrm{bt}}^{H}\right)w_{k}-MP. \end{array}$$

Note that $Z=MI_{M}-h_{\mathrm{bt}}h_{\mathrm{bt}}^{H}$ is a positive semi-definite matrix [26]. Therefore, (27) is a convex function.

Substituting (26) and (27) into (10), Problem (10) can be reformulated as

(28) $$\begin{array}{cc} \max_{W} & \rho \left(ℜ\left\{\bar{v}\bar{w}\right\}-{∥U\bar{w}∥}^{2}+{∥U1\bar{w}∥}^{2}\right)+\sum_{k=1}^{K}w_{k}^{H}Zw_{k}\\ s.t. & C1. \end{array}$$

Since $h_{\mathrm{br},k}=h_{b,k}^{H}+h_{r,k}^{H}\Theta H_{\mathrm{br}}$ and $h_{\mathrm{bt}}=g_{\mathrm{bt}}^{H}+g_{\mathrm{rt}}^{H}\Theta H_{\mathrm{br}}$, according to the expressions in (16)–(24) and (27), it can be observed that (28) is a function of W and Θ. Note that when Θ is fixed, Problem (28) is an SDP convex problem and can be efficiently solved by the CVX toolbox [28].

#### 3.2.2. Step 2: Optimizing Passive Beamforming Matrix at the RIS

For given W, Problem (9) can be simplified to

(29) $$\begin{array}{cc} \max_{\Theta } & \rho R_{s}+P_{b}\\ s.t. & C2. \end{array}$$

In (29), the available phase range of each reflective element depends on the bit of RIS. When W is fixed and power constraints are removed, the objective function in (29) regarding Θ still remains non-convex. Considering the complexity of the problem, here, we adopt a local search method [29] to solve it. For the optimization of the phase of the *n*-th RIS element, we first fix the phase values of the other *N*-1 RIS elements to their initial values (for those with already optimized phase values, these are fixed at their optimal values). We enumerate all possible values within the feasible region $\left\{\vartheta_{n}=\frac{2\pi i}{2^{b}},i=0,1,\ldots ,2^{b}-1\right\}$, identifying the value that maximizes the secrecy rate as the optimal phase value for the *n*-th element. Subsequently, we continue the search process until we obtain the optimal phase values for all RIS elements. The searching process will be executed $N\times 2^{b}$ times until all RIS elements obtain the optimal phase values.

#### 3.2.3. Overall Optimization Framework

In Section 3.2.1 and Section 3.2.2, we have developed solutions to the problems of BS beamforming optimization and RIS phase shift optimization. In this section, we will describe the proposed overall alternate optimization scheme. Specifically, we execute Step 1 and Step 2 sequentially, and then alternatively iterate between these two steps to facilitate a RIS-assisted secure ISAC, as shown in Algorithm 1. In the algorithm, Line 1 is to optimize the variable settings for feasible initial values. Lines 3–11 involve alternately solving for W and Θ based on the convex problems transformed in (10) and (29). In this context, Lines 3–6 pertain to various auxiliary variables involved in the optimization process.
**Algorithm 1** Proposed joint beamforming scheme in RIS-assisted secure ISAC systems  1:**Input** initial values of $W^{\left[0\right]}$, $\Theta^{\left[0\right]}$, $\beta_{k}$, $\varepsilon_{k}$, $\beta_{r,k}$, $\varepsilon_{r,k}$, and the number of quantization bits *b*; set the initial iteration number i=1;  2:**Repeat**  3: Calculate $\beta_{k}^{\left[i\right]}$ by solving (14);  4: Update $\varepsilon_{k}^{\left[i\right]}$ by solving (15);  5: Calculate $\beta_{r,k}^{\left[i\right]}$ by solving (21);  6: Update $\varepsilon_{r,k}^{\left[i\right]}$ by solving (22);  7: Given $\Theta =\Theta^{\left[i-1\right]}$, update $W^{\left[i\right]}$ by solving (10);  8: Given $W=W^{\left[i\right]}$, update $\Theta^{\left[i\right]}$ by solving (29);  9: For n=1 to *N* do10:  Assign all possible values to $\vartheta_{n}$ and select the value maximizing the $R_{s}$ in (29);11:  End For12: *i* = *i* + 113:**Until**$\left|R_{s}^{\left[i-1\right]}-R_{s}^{\left[i\right]}\right|\leq \delta$ or maximum iteration reached.

The computational complexity of Algorithm 1 is analyzed next. We can observe that the complexity of the algorithm is primarily concentrated in lines 7 and 8. Line 7 involves solving an SDP problem; its computational complexity for one iteration is $O\left(M^{4.5}\right)$. Line 8 is used to solve the optimization of discrete phase shift performs for Algorithm 1, with the respective computational complexity of $O\left(N\times 2^{b}\right)$. Therefore, the overall computational complexity of Algorithm 1 is in the order of $O\left(I_{t}\left({KM}^{4.5}+N\times 2^{b}\right)\right)$, where $I_{t}$ denotes the iteration times. The convergence of Algorithm 1 will be demonstrated in the subsequent section.

## 4. Simulation Results

In this section, we present simulation results to validate the performance of the RIS-assisted secure ISAC system. We consider the RIS-assisted secure ISAC system depicted in Figure 1. Assume the direct link channel follows Rayleigh fading and the RIS-aided channels follow Rician fading. In the simulation, we set the number of antennas at the BS as M=32, employing a ULA with half-wavelength spacing between adjacent antennas and the total power budget as P=20 dBm, $\sigma_{k}^{2}=-90$ dBm, $\sigma_{t}^{2}=-90$ dBm, and $\delta =10^{-3}$. The Rician factor is four, and the regularization parameter is 100. *K* single-antenna users are uniformly and randomly distributed within a circle centered at (200 m, −50 m) with a radius of 30 m. The BS and the RIS are located at (0 m, 0 m) and (200 m, 0 m), respectively. The target is positioned at an azimuth angle of $\theta_{\mathrm{bt}}=45^{\circ }$ and $\theta_{\mathrm{rt}}=45^{\circ }$. The path loss models for direct and indirect links are $\mathrm{Los}=32.6+36.7\log_{10}\left(di\right)$ dB, $\mathrm{NLos}=35.6+22\log_{10}\left(di\right)$ dB according to [30], where di is the link distance.

**Figure 2:**
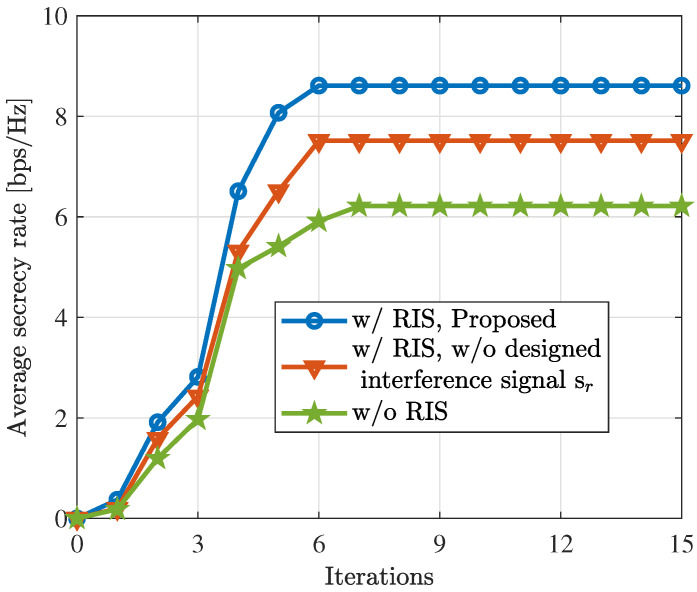
Convergence of the proposed algorithm for scenarios with RIS, without RIS, and without the designed interference signal. ‘w/o’ and ‘w/ ’ stand for without and with, respectively.

Before showing the ISAC performance, we demonstrate the convergence of the proposed Algorithm 1. Figure 2 illustrates the convergence of the proposed algorithm with RIS, without RIS, and without the designed interference signal, where N=20. As clearly shown, the average secrecy rate achieved by the proposed algorithm exhibits rapid growth with an increasing number of iterations, reaching convergence in less than 10 iterations. We also see that the introduction of RIS and the designed interference signals significantly enhance the security performance of the ISAC system.

**Figure 3:**
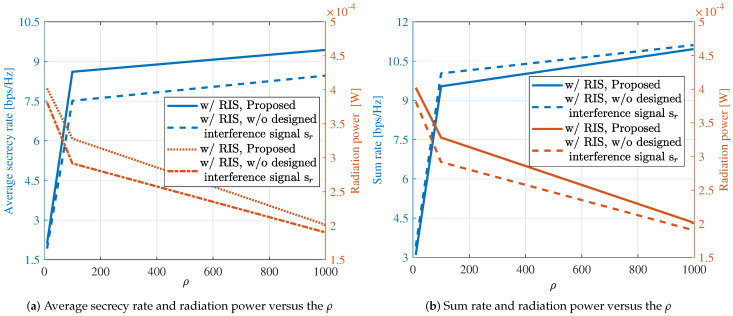
Performance trade-off.

Figure 3 illustrates the impact of the regularization factor ρ introduced in Problem (9). It can be observed that the proposed method converges for different values of ρ. However, due to the different weighting of the two physical quantities in (9) by ρ, we can see that the performance achieved by the ISAC system varies with different regularization factors. Figure 3a and Figure 3b, respectively, depict the achieved communication secrecy rate, sum rate, and sensing radiation power under different values of ρ. Across various regularization factors, the system’s achievable sum rate consistently surpasses the secrecy rate. This implies that maximizing the secrecy rate does not adversely affect the overall sum rate of the system. And from the simulation results, it can be seen that the performance achieved by the ISAC system varies with different regularization factors, which further proves the rationality of our proposed method.

**Figure 4:**
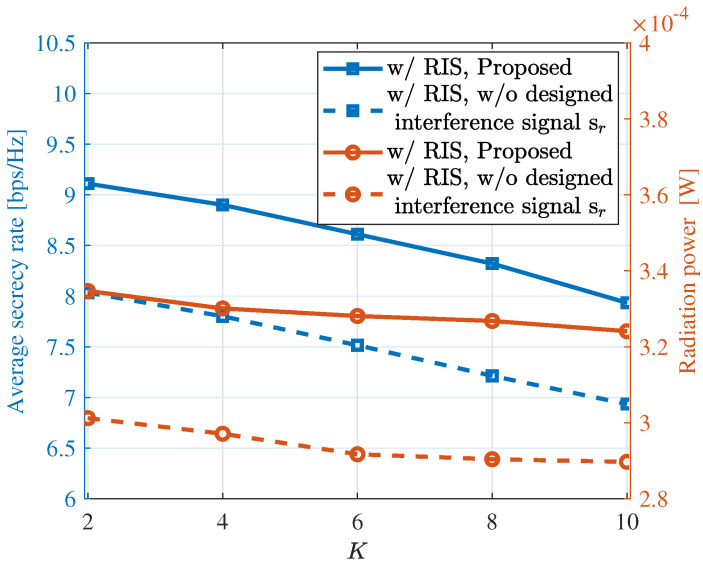
The communication and sensing performance under different values of *K* (user number), where curves with circle markers use the right y-axis.

In Figure 4, we evaluate the secrecy rates and radar radiation power of the considered ISAC system under different network densities, as indicated by the numbers of legitimate users in a confined region. It can be observed that as the number of users increases, the two performance metrics slightly degrade. This is reasonable due to the increasing channel coherence. However, it is worth noting that the proposed approach actively transmitting interference signals achieves non-trivial performance improvements over the considered range of *K*s. Specifically, when the number of legitimate users is six, the ISAC system with interference signals exhibits approximately a 13% improvement in the secrecy rate and an 11% increase in radiation power compared to the ISAC system without interference signals. This validates that introducing interference signals can effectively enhance the performance of the ISAC system. Moreover, our approach exhibits robust performance across different numbers of users, further confirming the robustness and applicability of the proposed method.

**Figure 5:**
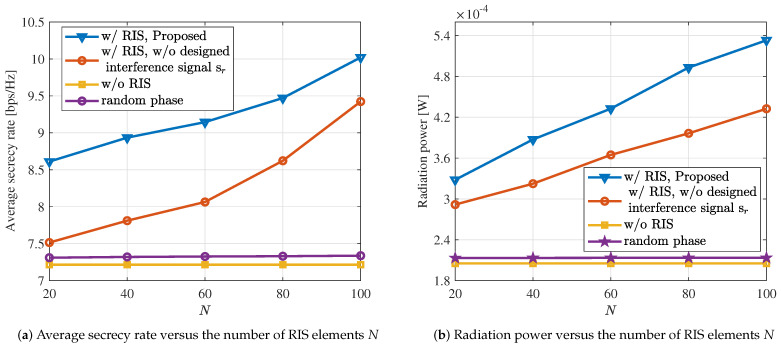
The communication and sensing performance under different values of *N*.

In Figure 5a, we compare the average secrecy rate with the number of RIS elements *N*. We can see that the performance of all schemes, except for the case without RIS, increases with the increment of *N*. Furthermore, compared to the method without the designed interference signals, the proposed approach achieves significant performance improvement. Specifically, when N=60, the average secrecy rate of the system increases by 15%. Similarly, in Figure 5b, we compare the radiation power with the number of RIS elements *N*. It can be observed that the radiation power gradually increases with the increment of *N*. At N=60, the system’s radiation power increases by 18%. Additionally, we find that the proposed approach exhibits a significant improvement in sensing performance. Combining the results from Figure 5a,b, the proposed algorithm significantly enhances the performance of the ISAC system. The introduction of RIS and interference signals has significantly enhanced the ISAC system’s performance.

**Figure 6:**
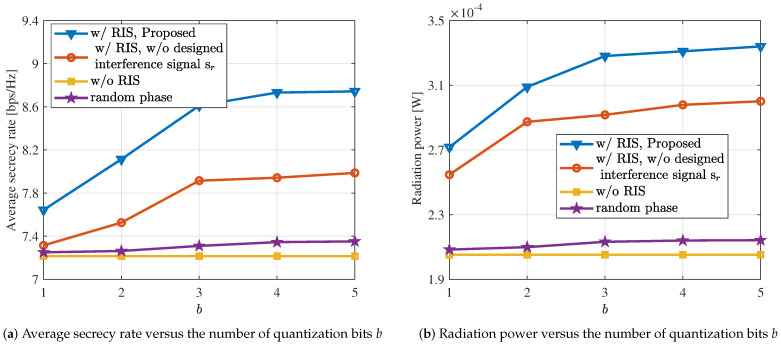
The communication and sensing performance under different values of *b*.

In Figure 6, the achievable average secrecy rate and radiation power of the system are plotted against the increasing bit quantization number, ranging from 1 to 5. In Figure 6a, we can observe a clear advantage of the proposed approach in terms of secrecy rate. In the presence of interference signals, the average secrecy rate can be increased by up to 10%. Additionally, we observed that the bit quantization number increases from 1 to 4, and then from 4 to 5; the average secrecy rate remains relatively constant. This suggests that the system’s performance reaches a saturation point once the quantization bit number exceeds 4. Similarly, in Figure 6b, we can also observe a significant increase in radiation power with the addition of RIS and the introduction of interference signals. Likewise, as the quantization bit number varies, a phenomenon similar to Figure 6a is evident.

**Figure 7:**
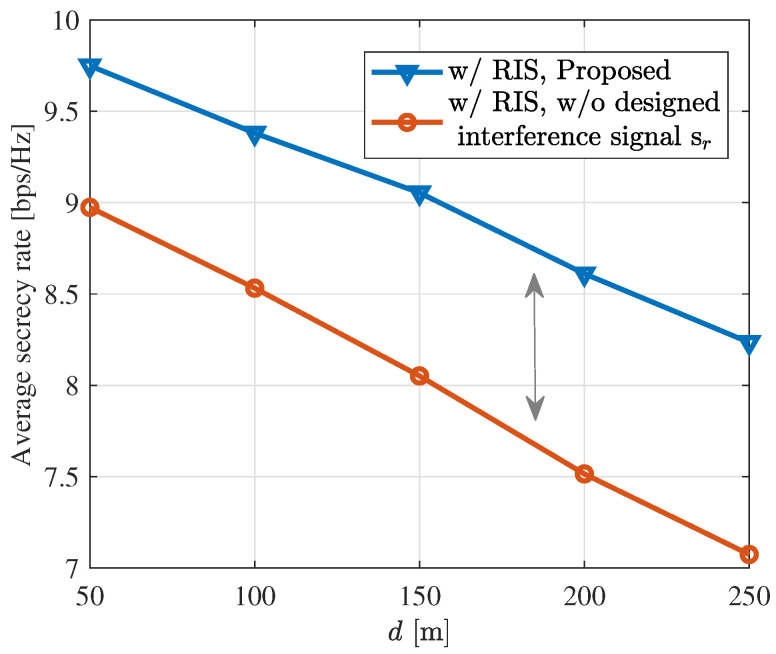
Average secrecy rate versus the horizontal distance of the RIS from the BS *d*.

Figure 7 illustrates the average secrecy rate of legitimate users when N=20, while moving the RIS from (50 m, 0 m) to (250 m, 0 m). It can be observed that as the distance between RIS and BS increases, the ISAC system’s secrecy rate decreases and its security performance is weaker. Additionally, we also observe that under the same power constraints, introducing interference signals can provide a better secrecy rate.

## 5. Conclusions

In this paper, we introduced the design of a secure ISAC system, proposing a joint optimization scheme on the transmit beamforming and the RIS coefficient matrix. Specifically, leveraging the potential of the RIS, we added the designed interference signals at the BS transmitter to confuse eavesdroppers attempting to intercept information from legitimate users. To ensure both the secrecy rate for legitimate users and the sensing performance of target detection, we formulated an optimization problem aiming to maximize the secrecy rate for legitimate users and the sensing radiation power. To overcome the non-convexity of the optimization problem, techniques such as FP and SDR were applied to convert the non-convex problem into a convex one. The problem was then solved using a low-complexity alternating optimization approach. Simulation results validated the effectiveness of the proposed method, demonstrating that the inclusion of RIS and interference signals can effectively enhance the ISAC system’s secrecy rate and sensing performance.

Though the optimization techniques applied in this work have achieved non-trivial performance enhancements in the considered secure ISAC system, it can be an interesting future work to further explore other optimization techniques, such as the popular majorization–minimization (MM) [24] and the manifold optimization [31]. In the future, we may consider exploring new methods to control nulls in the transmission waveform design to enhance the security rate and energy efficiency of the ISAC system [32]. And machine learning methods are showing promising results in addressing the issue of channel correlation between eavesdroppers and legitimate users [33], and in the future, we can further leverage machine learning techniques to enhance the security and efficiency of ISAC systems. Additionally, extending the proposed scheme to multi-RIS scenarios can be an interesting future work.

## Data Availability

The data presented in this study are available on request from the corresponding author. The data are not publicly available due to legal restrictions.
